# Heterogeneity in subnational mortality in the context of the COVID-19 pandemic: the case of Belgian districts in 2020

**DOI:** 10.1186/s13690-022-00874-7

**Published:** 2022-05-06

**Authors:** Benjamin-Samuel Schlüter, Bruno Masquelier, Carlo Giovanni Camarda

**Affiliations:** 1 IACCHOS (DEMO), Catholic University of Louvain (UCLouvain), Louvain-la-Neuve, Belgium; 2 Mortality, Health and Epidemiology, Institut National d’Etudes Démographiques (INED), Paris, France

**Keywords:** Covid19, Mortality, Subnational, Small-area, Belgium, Bayesian models

## Abstract

**Background:**

The COVID-19 pandemic has led to major shocks in mortality trends in many countries. Yet few studies have evaluated the heterogeneity of the mortality shocks at the sub-national level, rigorously accounting for the different sources of uncertainty.

**Methods:**

Using death registration data from Belgium, we first assess change in the heterogeneity of districts’ standardized mortality ratios in 2020, when compared to previous years. We then measure the shock effect of the pandemic using district-level values of life expectancy, comparing districts’ observed and projected life expectancy, accounting for all sources of uncertainty (stemming from life-table construction at district level and from projection methods at country and district levels). Bayesian modelling makes it easy to combine the different sources of uncertainty in the assessment of the shock. This is of particular interest at a finer geographical scale characterized by high stochastic variation in annual death counts.

**Results:**

The heterogeneity in the impact of the pandemic on all-cause mortality across districts is substantial: while some districts barely show any impact, the Bruxelles-Capitale and Mons districts experienced a decrease in life expectancy at birth of 2.24 (95% CI:1.33–3.05) and 2.10 (95% CI:0.86–3.30) years, respectively. The year 2020 was associated with an increase in the heterogeneity of mortality levels at a subnational scale in comparison to past years, measured in terms of both standardized mortality ratios and life expectancies at birth. Decisions on uncertainty thresholds have a large bearing on the interpretation of the results.

**Conclusion:**

Developing sub-national mortality estimates taking careful account of uncertainty is key to identifying which areas have been disproportionately affected.

**Supplementary Information:**

The online version contains supplementary material available at (10.1186/s13690-022-00874-7).

## Background

The COVID-19 pandemic has not only halted progress in life expectancy but also brought about an abrupt drop in most Western countries, with reductions of more than a year documented in 11 countries in males and 8 countries in females in 2020 [1]. As of 31 December 2020, more than 1.9 million COVID deaths had been reported to the WHO [2], and the Institute for Health Metrics and Evaluation (IHME) estimated that the pandemic has resulted in more than 5 million COVID deaths in total, after factoring in estimated unreported deaths [3]. Between the onset of the pandemic and the 31 December 2020, Belgium has been hit hard by the pandemic with 19,846 deaths according to Sciensano [4]. The country was ranked 17^th^ worldwide according to deaths per 100,000 population [5]. Tallies of deaths directly attributed to the virus, despite being informative, are influenced by variations in testing capacity and definitional inconsistencies in the counting of COVID-19 deaths [6, 7], making comparison across countries unreliable. As a consequence, all-cause mortality has been widely used to accurately measure the pandemic’s impact on mortality using excess deaths [8, 9]. This indicator is defined as the difference between the observed number of deaths and what would have been expected had the pandemic not happened. There is a long tradition of using excess deaths to assess the death toll of pandemics and other extreme events [10]. Despite broad consensus on its use [8], this measure is not perfect as it mixes the direct effect of the pandemic on all-cause mortality with the indirect effects, such as those due to a shortage of health services for other medical emergencies, reductions in traffic accidents and influenza deaths in response to lockdowns and other measures of social distancing, or even the economic slowdown. This has led some authors to consider both excess mortality and direct COVID-19 deaths [1, 11]. Other researchers have used changes in life expectancy as a metric to compare countries, regions, or time periods [1, 12, 13]. The main advantages of this synthetic indicator are that it is a commonly used metric, is insensitive to variations in age structures, and is expressed in years [14]. The magnitude of the shock can be assessed in the same way as excess mortality, comparing observed levels of life expectancy in 2020 against estimates had the pandemic not happened, obtained from a counterfactual scenario; however, it is usually assessed by comparing life expectancy in 2020 to that of previous years [1, 13]. Such comparisons often fail to account for the increasing trend in life expectancy observed in most Western countries [15], resulting in underestimates of the impact of the pandemic on mortality. Accounting for the secular trend would require projecting mortality for 2020 and propagating the uncertainty associated with the projection into the assessment of the shock introduced by the pandemic on life expectancy.

Considering the difficulties associated with assessing the mortality shock nationally, relatively few studies have evaluated the heterogeneity of the mortality shock at a small-area level [12, 13, 16, 17]. Yet national-level analyses may conceal substantial heterogeneity in how various small areas have been affected. Small-area estimation is required to identify districts that might be more vulnerable to potential next waves or other future pandemics. Assessing the mortality change on a finer geographical scale comes with at least two additional challenges. First, such assessment requires working with smaller populations where annual death counts are subject to higher stochastic variation [18]. While this has a direct impact on uncertainty around computed life expectancy, this is usually not accounted for at a small-area level (see [13] as an example). Second, obtaining the counterfactual scenario for 2020 requires projecting life expectancy for 2020 at the district level, whereas such projections are commonly performed at the national level.

In this article, we propose a methodology to assess how mortality in various Belgian districts has been affected in the context of the pandemic. Districts correspond to NUTS 3 in Belgium, the finest geographical scale used by Eurostat. With this method, we address two related research questions:

- How has the pandemic affected subnational heterogeneity in mortality levels across Belgian districts?
- What is the magnitude of the shock at the district level, accounting for the various sources of uncertainty?

First, we estimate standardized mortality ratios (SMRs) using a Bayesian hierarchical model (BHM). The hierarchy enables the change in SMR heterogeneity over years to be estimated by assuming that, on a yearly basis, the SMRs all come from the same distribution. In addition, SMRs are not affected by the population age structure and only require total deaths instead of age-specific mortality rates at the district level, which reduces noise. Second, we use life expectancy at birth to assess the magnitude of the shock on district-level mortality in 2020. This indicator summarizes the mortality intensity across all ages, without being affected by the different population structures of the districts. We propose a method, inspired by Ševčíková and Raftery (2021) [19], for projecting district life expectancy for 2020 while accounting for all sources of uncertainty. We then compare these estimates with what has been observed, while also accounting for uncertainty in life expectancy computation due to small population sizes at the district level. Finally, we highlight how important it is to use different uncertainty thresholds when performing analyses at a small-area level.

## Methods

### Data sources

We employ aggregate-level data from the Belgian national civil register. Belgium has several administrative levels, 3 regions (Wallonia, Flanders and Bruxelles) 10 provinces, 43 districts and 581 communes. Here we focus on districts, ranging in population size from approximately 38,000 to 1.2 million. Our observations consist of deaths $D^{d,t}_{x}$ and person-years of exposure $N^{d,t}_{x}$ for district *d*, year *t* and age group *x*∈ {0,1-4, 5-9,...,90-94,95+}. Setting 95+ as the upper bound for age reduces noise in the data for the oldest age range in the context of a small-area analysis. Exposure consists of mid-year populations. The mortality rate at age *x* for district *d* and year *t* is thus expressed as $M^{d,t}_{x}=\frac {D^{d,t}_{x}}{N^{d,t}_{x}}$.

### Standardized mortality ratio and heterogeneity

The SMR is widely used for indirect standardization [20]. Here it consists of the ratio of district deaths to the number of deaths that would be expected if the district had experienced the national age-specific mortality rates of the selected year. For district *d*, it can be expressed as follows:

$$\begin{aligned} SMR_{d} = \theta_{d} & = \frac{\sum_{x=0}^{95}M^{d}_{x}N^{d}_{x}}{\sum_{x=0}^{95}M^{Nat}_{x}N^{d}_{x}} = \frac{\sum_{x=0}^{95}D^{d}_{x}}{\sum_{x=0}^{95}M^{Nat}_{x}N^{d}_{x}} \\ & = \frac{Deaths_{d}}{Expected~deaths_{d}} \,, \end{aligned} $$

where, for simplicity we removed indices for year and superscript *Nat* corresponds to national level. For a given year and district, deaths can then be modelled as follows

$$Deaths_{d,t}| \theta_{d,t} \sim Poisson(\theta_{d,t} \times Expected\ deaths_{d,t}) $$

where *θ*_*d*,*t*_ is the SMR we want to estimate for district *d* and year *t*. The Poisson distribution is apt as it allows the uncertainty to vary with the size of the district’s population. For each year in the period 2015-2020, we model *θ*_*d*_ ∀*d*∈{1,2,3,..,43} as coming from a single distribution allowing districts to borrow strength from each other and shrinking estimates for smaller districts towards the mean. We further assume that all yearly averages, *μ*_*θt*_, share the same distribution, centered in *M*_*θ*_. The standard deviations of SMRs, *σ*_*θt*_, are estimated independently for each year. We use the following weakly informative priors [21]:

$$\begin{aligned} \theta_{d,t} & \sim Normal^{+}(\mu_{\theta t}, \sigma_{\theta t}) \\ \mu_{\theta t} & \sim Normal^{+}(M_{\theta}, 1) \\ M_{\theta} & \sim Normal^{+}(1, 1) \\ \sigma_{\theta t} & \sim Normal^{+}(1, 1) \end{aligned} $$

The posterior distribution of *σ*_*θt*_ for each year is of particular interest as it consists of the estimated standard deviation of the SMR distribution. Hence, by comparing its median and credible interval, the heterogeneity in SMR values in 2020 in comparison to previous years can be assessed.

As the SMR is a measure of mortality relative to the national level, we also need an absolute measure of the shock in the context of the COVID-19 pandemic.

### Life expectancy as a measure of the shock to mortality

In order to assess how mortality rates have been affected during the COVID-19 pandemic at the district level, we will use life expectancy at birth (*e*^0^) computed without stratification by sex, which we simply refer to as life expectancy. To accomplish this, we need two pieces of information. First, we need a projection of *e*^0^ at the district level in 2020. This forecast value can be seen as a counterfactual scenario, had the COVID-19 pandemic not happened. Second, we need the observed *e*^0^ at the district level in 2020, obtained from standard life table techniques. We account for uncertainty around the observed life expectancy values using Chiang’s method [22]. Computing the difference between the projected and observed estimates, combining their associated uncertainty, allows us to adequately assess the magnitude of the shock on district-level mortality in 2020.

#### Probabilistic projections of district life expectancy

The methodology that we developed to obtain projections at the district level builds on recent work from Ševčíková and Raftery (2021) [19].

Let $e^{0}_{Nat,t}$ be national life expectancy at year *t*. We define life expectancy in district *d* at year *t*, $e^{0}_{d,t}$, as a function of the national level using

$$ e^{0}_{d,t} = e^{0}_{Nat,t} + \alpha_{d,t} \,, $$

where *α*_*d*,*t*_ can be viewed as the gap between district and national life expectancy at year *t* and will be treated as a stochastic variable.

We first project life expectancy in 2020 at the national level using the Lee–Carter model [23] estimated over the period 1991–2019. Estimating the model using a Bayesian framework means we can produce a set of trajectories from its posterior predictive distribution, $e^{0}_{Nat,2020,i}$ for trajectory *i*.

District-specific deviations are modelled using a Bayesian hierarchical linear model:

$$\alpha_{d,t}| \mathbf{\beta_{d}}, \sigma_{\alpha d} \sim Normal(\beta_{0, d} + \beta_{1, d} \cdot t, \sigma_{\alpha d}) \,, $$

over the period 1991–2019. The linearity over time approximates the data quite well (Additional Figs. S1-S3). This can be explained by the fact that over the period 1991–2019, the health performance of a district, in comparison to the national performance, is usually stable over time, or showing a constant trend. The hierarchical structure of the model means that betas for smaller districts are informed by all other districts, leading to shrinkage in estimation. This is of interest as smaller districts show higher variation in *α*_*d*,*t*_ (the case of Marche-en-Famenne in Additional Fig. S3). Weakly informative priors defined for intercepts (*β*_0,*d*_) and slopes (*β*_1,*d*_) can be found in Additional file 1. We tested the sensitivity of our results to the priors.

Variance for *α*_*d*,*t*_ denoted by *σ*_*αd*_ is allowed to vary across districts because fluctuations in *α*_*d*,*t*_ depend on district’s population size (Additional Fig. S3). This uncertainty will thus be propagated into the projection for each district.

Finally, we simulate the *i*-th trajectory of *α* for district *d* in 2020, *α*_*d*,2020,*i*_, as follows:

$$ \alpha_{d,2020,i} \sim Normal(\beta_{0, d, i} + \beta_{1, d, i} \cdot 2020, \sigma_{d, i}) \,. $$

This step allows to account for both the parameters uncertainty and variation in the data. The *i*-th trajectory from the posterior predictive distribution of district life expectancy in 2020 is given by

$$ e^{0}_{d,2020,i} = e^{0}_{Nat,2020,i} + \alpha_{d,2020,i} \,, $$

where $e^{0}_{Nat,2020,i}$ is the *i*-th trajectory from the national life expectancy being projected. Consequently we account for the uncertainty in the national projection as well as in the estimation of the district-specific deviations.

#### Observed life expectancy and its uncertainty in 2020

In order to reflect uncertainty in life-table computation at the district scale, we applied the Chiang method [22]. Let _*n*_*q*_*x*_ and _*n*_*D*_*x*_ be the probability of dying from the 2020 observed life table and observed deaths in a given district, respectively. For each age-group [*x*,*x*+*n*], we assume that deaths are realizations from a binomial distribution where the number of trials is equal to the people at risk computed as $\frac {{~}_{n}D_{x}}{{~}_{n}q_{x}}$ and the success probability for each trial is the probability of dying _*n*_*q*_*x*_. We can thus simulate an *i*-th series of death counts as follows:

$$ {~}_{n}D_{x,i} \sim Binomial\left(\frac{{~}_{n}D_{x}}{{~}_{n}q_{x}}, {~}_{n}q_{x}\right) \,. $$

We repeat this procedure 4,000 times for each district obtaining 4,000 associated life tables and life expectancies. We combine these outcomes with 4,000 posterior trajectories from the projection exercise. This allows us to compute, for each district, a distribution of differences *Δe*^0^ between observed and counterfactual life expectancy in 2020 that accounts for uncertainty coming from both observed and forecast life expectancy for that year. Quantities such as the medians and quantiles of these distributions provide point estimates and credible intervals for the difference between observed and projected districts’ *e*^0^, and hence an assessment of the shock on mortality. Analyses were run with the R software version 4.0.3. We used STAN software [24] and rstan package [25]. All the R codes used to perform the analysis are available on Github: https://github.com/benjisamschlu/Subnationalcovid19.

## Results

### Standardized mortality ratios and heterogeneity

**Fig. 1:**
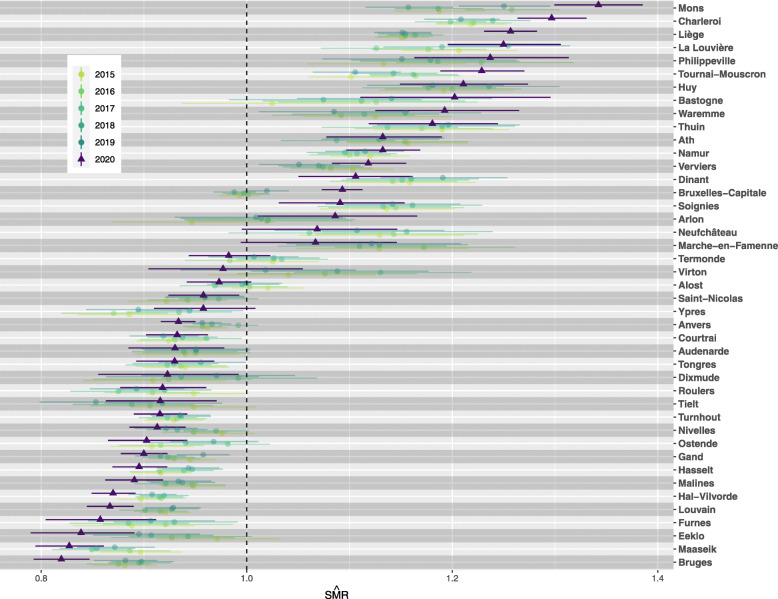
Estimated standardized mortality ratios with a 95% credible interval by Belgian district, 2015–2020

Figure 1 shows the annual estimates of SMR for each district over the period 2015–2020, with their associated 95% credible intervals. An SMR equal to 1 means that the observed deaths in the district equal the expected deaths if the population in the district had experienced the national age-specific mortality rates, while an SMR above (or below) 1 reflects more (or fewer) deaths than expected compared to national mortality. At the district level, we observe temporal variation in SMR estimates. For example, over the years Mons and La Louvière- characterised by a higher level of mortality relative to the national level- have SMRs ranging between 1.16 (95% CI:1.12–1.20) and 1.26 (95% CI:1.21–1.30) and 1.13 (95% CI:1.07–1.18) and 1.25 (95% CI:1.20–1.31), respectively. Other districts such as Liège, Audenarde, Turnhout, Namur, Hal-Vilvorde, Soignies, Bruges, Malines, Louvain, Bruxelles-Capitale, Charleroi and Hasselt show little temporal variation in their SMR between 2015 and 2019. Spatial heterogeneity in districts’ SMR is present before 2020, reflecting disparity in mortality levels across Belgian districts. For example, the year 2019 is characterised by the largest ratio of highest to lowest SMR over the 2015-2019 period. During that year, La Louvière had a death count 25% (95% CI: 20–31) higher than expected had the district experienced the national mortality level. At the other extreme, Tielt had 15% (95% CI: 9–20) fewer deaths than expected under the national mortality level (see Table 1). However, 2020 seems to be characterized by even higher spatial variation in SMRs. Some districts have higher SMR values than in previous years: Mons 1.34 (95% CI: 1.30–1.39), Charleroi 1.30 (95% CI: 1.26–1.33), Liège 1.26 (95% CI: 1.23–1.28), Tournai-Mouscron 1.23 (95% CI: 1.19–1.27) and Bruxelles-Capitale 1.09 (95% CI: 1.07–1.11). Others have lower SMRs than in previous years: Bruges 0.82 (95% CI: 0.79–0.85), Maaseik 0.83 (95% CI: 0.79–0.86), Eeklo 0.84 (95% CI: 0.79–0.89), Louvain 0.87 (95% CI: 0.84–0.89), Hal-Vilvorde 0.87 (95% CI: 0.85–0.89), Malines 0.89 (95% CI: 0.86–0.92) and Anvers 0.93 (95% CI: 0.92–0.95). Table 2 presents posterior median estimates for the standard deviations of SMRs, *σ*_*θ*_, for each year in the 2015–2020 period. Through this table, we can assess the evolution in subnational SMRs’ standard deviation over the years. In 2020, the posterior distribution showed a clear shift to the right (see Additional Fig. S6), with an estimated standard deviation reaching 0.15 (95% CI: 0.12–0.19). This indicates a higher heterogeneity in districts’ mortality levels in comparison to previous years. This observation is also in line with an increase in the highest to lowest SMR ratio, reaching 1.64 in 2020 against 1.47 in 2019 (Table 1).

**Table 1:** Highest to lowest SMR ratio, 2015-2020

	Highest	Lowest	Ratio
2015	1.22 (Charleroi)	0.88 (Bruges)	1.38
2016	1.26 (Mons)	0.82 (Ypres)	1.44
2017	1.20 (Charleroi)	0.85 (Maaseik)	1.41
2018	1.24 (Charleroi)	0.85 (Maaseik)	1.45
2019	1.25 (La Louvière)	0.85 (Tielt)	1.47
2020	1.34 (Mons)	0.82 (Bruges)	1.64

**Table 2:** Estimated standard deviation of the SMRs (*σ*_*θ*_), 2015-2020

	Point estimate	95% CI
2015	0.11	0.09–0.15
2016	0.12	0.10–0.16
2017	0.11	0.09–0.14
2018	0.12	0.09–0.15
2019	0.12	0.10–0.15
2020	0.15	0.12–0.19

**Fig. 2:**
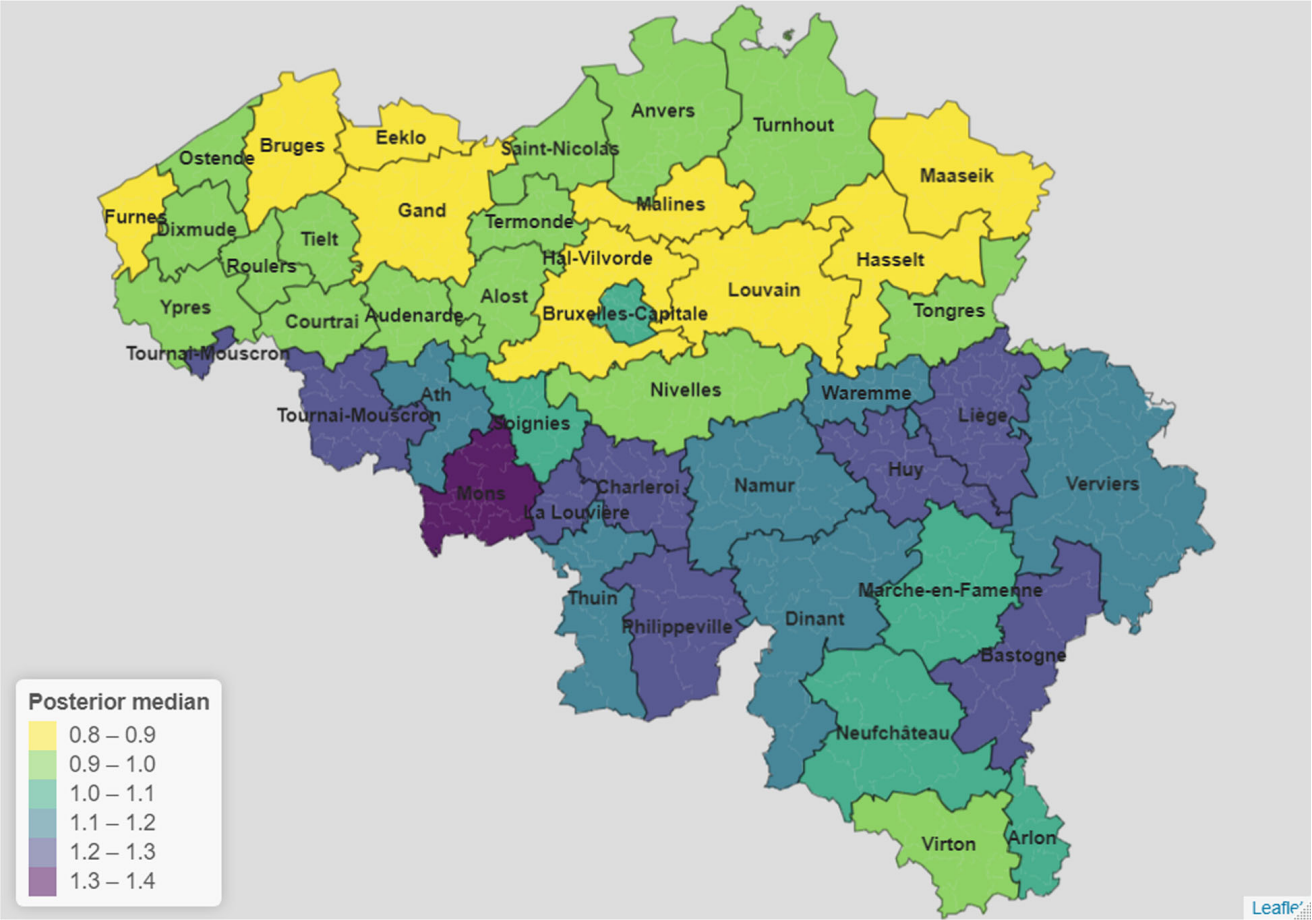
Estimated standardized mortality ratios by Belgian district, 2020

SMRs point estimates are displayed on a map for 2020 in Fig. 2. We see a clear North–South divide in mortality levels within the country, not particular to 2020. In fact, during the pandemic year, all districts belonging to the Walloon region have an SMR significantly above 1 except Neufchâteau, Marche–en–Famenne, Virton and Nivelles. Conversely, all districts belonging to the Flemish region have an SMR significantly below 1 except Alost and Termonde. In the past, the Bruxelles-Capitale district has faced mortality rates that were similar to the experience of Flanders, but in 2020, this district faced mortality rates closer to those witnessed in Wallonia.

**Fig. 3:**
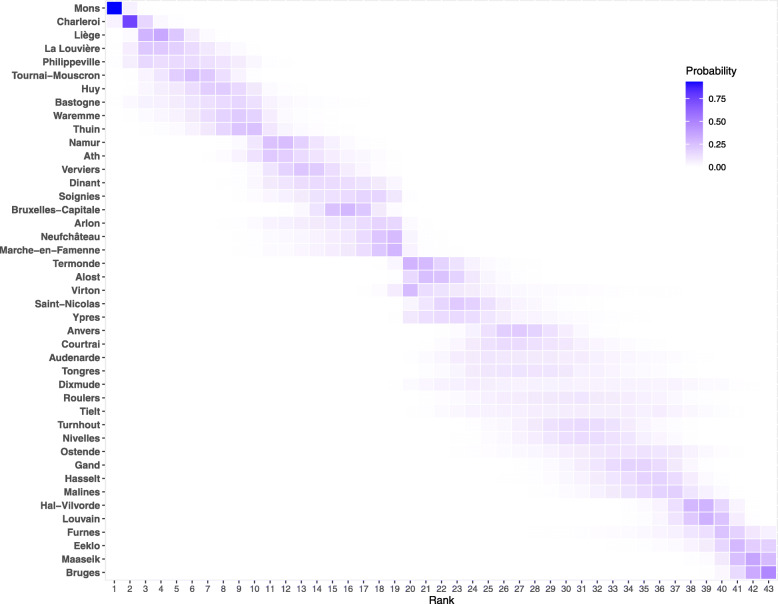
Posterior ranking probabilities of standardized mortality ratios at Belgian district level, 2020

In the context of subnational analyses, the ranking of units matters. Posterior ranking probabilities permit the ranking of districts according to their posterior expected rank while reflecting uncertainty in the ranking [26]. Figure 3 presents posterior ranking probabilities for each district in 2020. Each square in the figure represents the posterior probability that district *d* has rank *k*∈{1,2,3,…,43}. The darker the color the more a certain position in the rank can be expected for a given district, with the sum over the row equal to 1. The heat map shows a clear diagonal trend, leading us to conclude that ranking according to estimated SMR is rather certain, with Mons, Charleroi and Liège being the districts most likely to have experienced the highest mortality in 2020, while Bruges, Masseik and Eeklo experienced the lowest rates with respect to the national mean. Despite minor changes (most likely explained by random fluctuations in death counts for smaller districts), the ranking of districts has been stable over the years with the exception of Bruxelles-Capitale, which ranked significantly higher in 2020 relative to previous years.

### Difference between projected and observed districts’ life expectancies

**Fig. 4:**
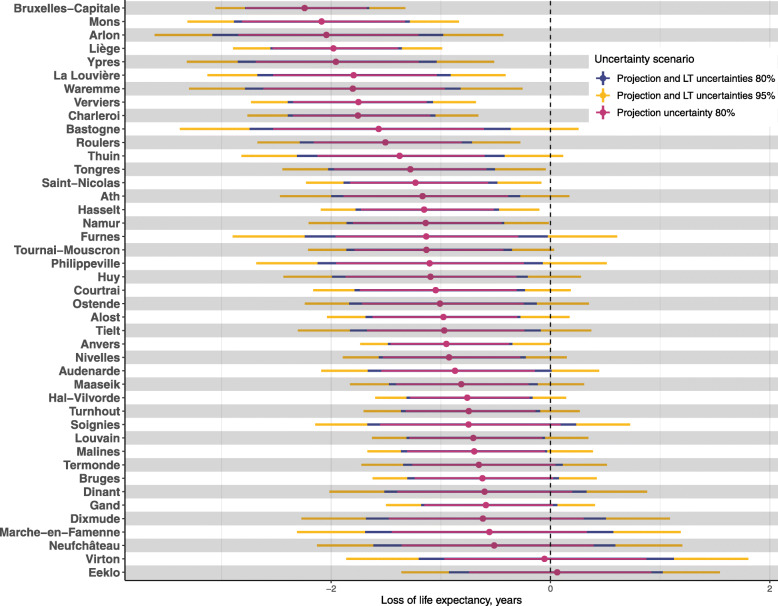
Differences between observed and projected life expectancy at district level in Belgium with uncertainty according to three scenarios, 2020

Moving on to our absolute measure of the shock, Fig. 4 shows the difference between the life expectancy observed in 2020 and the 2020 counterfactual projection with its associated credible intervals, for each district. Three credible intervals are presented: the red intervals correspond to the difference taken without accounting for uncertainty in the computation of life tables for the observed rates; the blue intervals account for uncertainty in such computation and set the credible interval at 80%; the yellow intervals account for uncertainty in such computation and set the credible interval at 95%. As an example, with respect to our counterfactual scenario, Bruxelles-Capitale, Mons, Arlon and Liège districts experienced a drop in life expectancy of 2.24 (95% CI:1.33–3.05), 2.10 (95% CI:0.86–3.30), 2.0 (95% CI:0.42–3.6) and 2.0 (95% CI:1.0–2.9) years, respectively. However, the width of the credible intervals varies according to the district’s population size, the sources of uncertainty considered and the level of uncertainty chosen. For instance in the case of Arlon, we see that not accounting for uncertainty in life-table computation based on the observed rates overestimates the precision of the shock assessment (the difference between the blue and red intervals). This can be observed for all districts having relatively small population sizes (Ypres, Bastogne, Furnes, Dixmude, Marche-en-Famenne, Neufchâteau and Virton). It is also worth noting that the widths of the 95% credible intervals for districts such as Furnes or Bastogne (with populations of 61,700 and 49,000 inhabitants, respectively) are close to three years of life expectancy. According to the most conservative credible interval (depicted in yellow), 14 out of 43 districts have had a statistically significant drop in their life expectancy in comparison to the projection. The magnitude of the drop differs across districts, however. When considering point estimates, all districts but one had a level of life expectancy in 2020 that was lower than expected based on the secular decline in mortality continuing had the pandemic not happened.

**Fig. 5:**
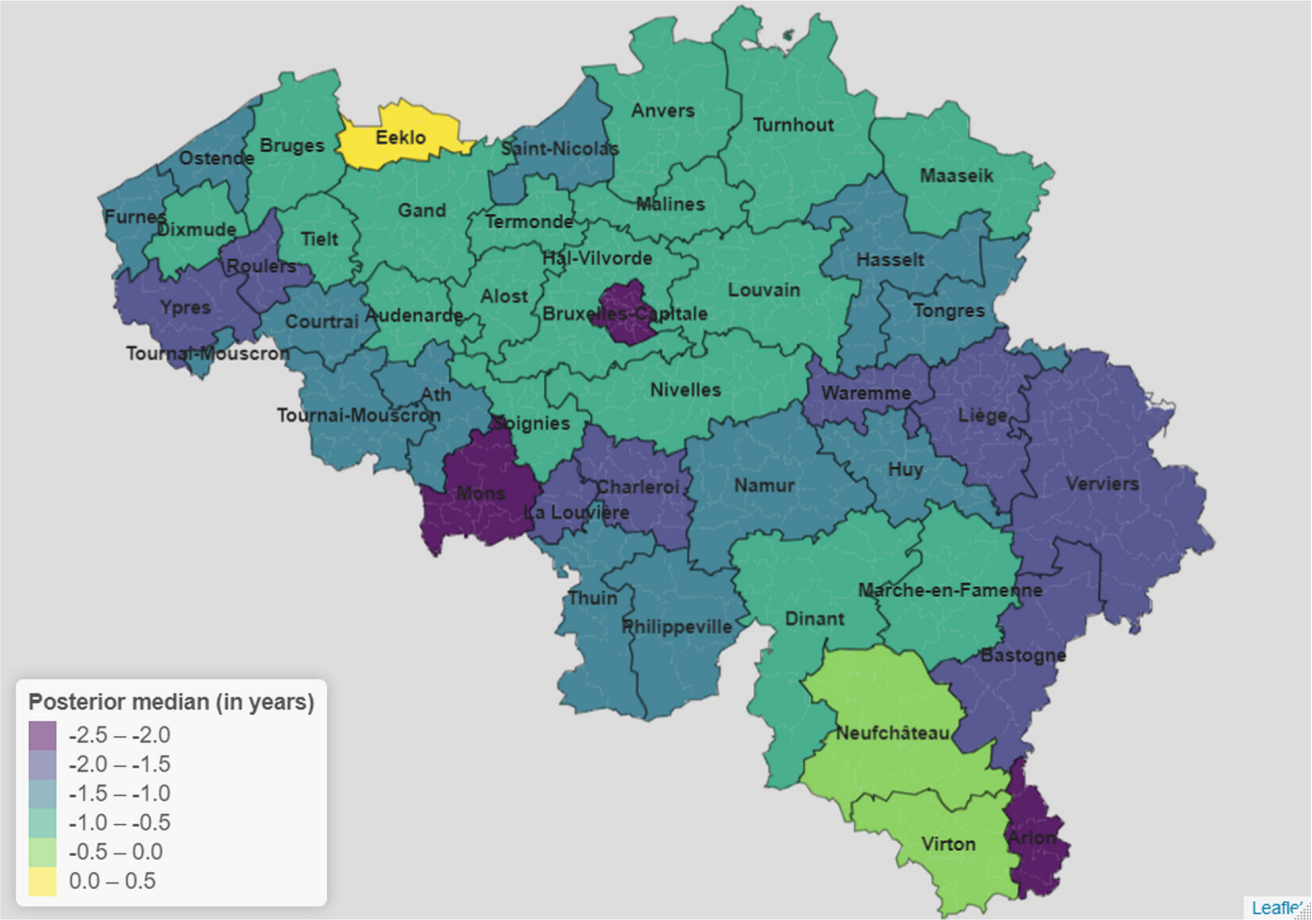
Estimated loss of life expectancy by Belgian district, 2020

We map the point estimates for loss of life expectancy in Fig. 5. In comparison to Fig. 2, the regional divide is less clear. In fact, out of the three districts experiencing the lowest estimated drop in life expectancy, two are in the South of Belgium (Virton and Neufchâteau). We nonetheless need to be careful as these districts are characterised by wide credible intervals. Districts at the border with Germany and Luxembourg where particularly affected (Arlon, Bastogne, Verviers and Liège). This seems to be also the case for Mons and Ypres, bordering France. Focusing on the center of Belgium, Bruxelles-Capitale seems to have been particularly affected in comparison to neighbouring districts.

It is important to note that the amount of uncertainty tolerated is highly determinant. Accounting for both sources of uncertainty and setting credible intervals at 80% leads us to conclude that only 11 districts did not experience a significant drop in life expectancy in 2020 compared to the projections. When only considering the uncertainty around the projection, all but 10 districts experienced a significant drop. In addition, the width of the credible intervals varies substantially depending on the uncertainty scenario chosen and the district’s population size. This points to the need for an assessment of uncertainty when computing subnational life expectancy at this level of spatial disaggregation.

**Fig. 6:**
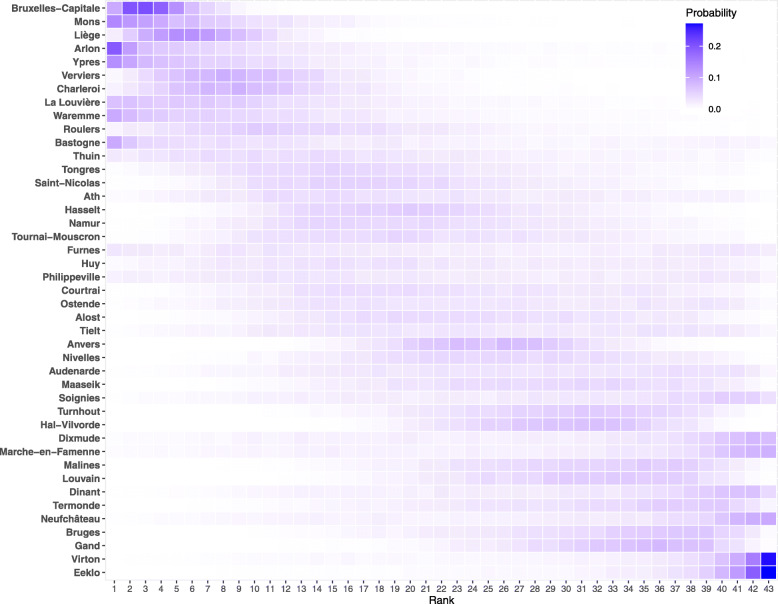
Posterior ranking probabilities of the differences between observed and projected life expectancy at district level in Belgium, 2020

Ranking districts according to their posterior expected differences between observed and projected life expectancies, *Δe*^0^, leads to high uncertainty in the ranking (see Fig. 6). This is a logical consequence of accounting for projection uncertainties in addition to uncertainty associated with life expectancy computation at the district level.

We also explored heterogeneity in life expectancy in 2020 relative to other years to consolidate our observation of increased variation in districts’ mortality levels for 2020. For each year and district combination, we obtained an associated 4,000 simulated life expectancies and computed the associated standard deviation across districts. For each year, we ended up with 4,000 bootstrapped $\phantom {\dot {i}\!}\sigma _{e^{0}}$. We transformed these into a density of $\phantom {\dot {i}\!}\sigma _{e^{0}}$ over the years (Additional Fig. S7). In line with previous observations of SMRs, we see that the density of $\phantom {\dot {i}\!}\sigma _{e^{0}}$ in 2020 has moved to the right, with its median equal to 1.72 (95% CI:1.63-1.81) years.

## Discussion

Our study proposed a methodology to assess how the mortality levels of various small-areas were affected in the context of the COVID-19 pandemic in 2020 using death registration data. Our application is on Belgian districts but the proposed method could be applied to other subnational contexts. We first computed SMRs over the period 2015–2020 and explored how their variation changed in 2020 compared to previous years. In addition, we compared observed with projected district life expectancies in 2020, accounting for different sources of uncertainty.

In 2020, three districts had deaths counts that were more than 20% higher than expected when assuming national mortality. At the other extreme, five districts had 10% fewer deaths than what would be expected assuming the national age-specific mortality rates. Our results are in line with the mortality surveillance at the provincial level realized by Sciensano [27]. In fact, they showed that the highest provincial SMRs were found in Bruxelles, Hainaut and Liège. Looking at Fig. 2, districts belonging to these provinces are indeed the ones characterised by higher SMR values.

We found that although heterogeneity in mortality levels was present before the pandemic, with a clear North–South divide (previously documented in [28], [29]), the shock to mortality in 2020 increased the variation in SMRs with respect to previous years. This is also confirmed by a higher heterogeneity in districts’ life expectancy at birth in 2020 relative to previous years. The increased spatial inequality of mortality can also be interpreted as a measure of the shock. When working at a subnational level, the goal of assessing which units were most affected is relevant for policy. With this in mind, the SMR metric proved suitable for ranking districts as it leads to greater certainty in the ranking. This was not the case when ranking districts according to their loss of life expectancy. Assessing the certainty in ranking is an important element of this study and should more systematically be done when assessing the shock of a pandemic at a small-area level.

For a given year, the SMR is expressed relative to the national mortality level and is thus not suitable for quantifying the magnitude of the shock on mortality in 2020. To achieve this, we compared the projected life expectancy with what has been observed, allowing us assess the magnitude of the shock at the district level, while accounting for the increasing trend in life expectancy. This exercise requires accounting for two sources of uncertainty: the uncertainty coming from projecting life expectancy at the district level in 2020, and the uncertainty in life table estimates based on age-specific mortality rates observed at the district level. The level of confidence we are willing to tolerate is thus determinant in the interpretation of our results. Indeed, the width of the intervals highly depends on the sources of uncertainty accounted for and the confidence level chosen. Choosing intervals of 95% and accounting for both the observed and projected uncertainties in life expectancies, we concluded that 14 districts experienced a significant drop in their life expectancy in comparison to their projections. The biggest drops were 2.24 in Bruxelles-Capitale (the capital city), 2.10 in Mons and 2 years in Arlon and Liège. However, when set- ting the interval threshold at 80% and not accounting for uncertainty around life table estimates for small areas, 33 districts out of 43 experienced a significant drop. Other research found capital cities to be areas within countries that were the most affected in 2020 [12, 13, 16]. Stockholm, the capital city of Sweden, experienced a drop of 1.5 year in its life expectancy between 2019 and 2020 [17]. In Mexico City, males lost about 6 years of life expectancy due to Covid-19, the biggest drop observed across all Mexican states for males in 2020 [13]. In Spain, Madrid lost 2.8 and 2.1 years of life expectancy between 2019 and 2020 for men and women, respectively [12]

These orders of magnitude seen at a small-area level are higher than the ones found by Aburto and colleagues (2021) [1] at the national level. In their study, they showed that Belgium saw a drop of approximately 1 year in life expectancy at birth for both males and females between 2019 and 2020. This difference in magnitude is expected when looking at the most affected districts within Belgium. This also need to be put in context of a world life expectancy estimated to have declined by -0.92 years between 2019 and 2020 [30].

Looking across districts, it is clear that the shock on mortality has been highly heterogeneous within the country, with some districts not showing any significant difference between observed and projected life expectancy. This is in line with the Belgian mortality surveillance showing that some provinces were not significantly affected according to their SMRs [27]. Other research also found significant differences in the magnitude of the shock [13, 16, 31]. For example, Trias-Llimós and colleagues [12] found 7 out of 17 and 5 out of 17 regions in Spain not showing any significant difference in annual life expectancy between 2019 and 2020 for men and women, respectively. At the other extreme, they showed that 5 regions experienced a drop higher than 1 year for both men and women. During the first wave in Italy, the northern regions witnessed more deaths relative to southern regions [32]. North-Eastern French departments also experienced higher deaths counts per 100,000 persons [33].

The observed subnational heterogeneity of the shock on mortality may stem from an unequal distribution of specific characteristics across districts. An important indicator that might play a role is the number of people in care homes present within a district [34, 35]. Indeed, care homes were strongly affected by the first wave [36] as they were not sufficiently prepared and hosted a majority of frail individuals. Between March and June 2020, 64% of Covid-19 deaths in Wallonia were residents in care homes [36]. The number of care homes thus may substantially increase the death toll at the district level. A high intensity of mobility within a district may also lead to higher transmission of the virus [37], with districts hosting more economic activity or international organizations being more at risk. This is in line with our observation that districts at the French, German and Luxembourg borders seemed to experienced higher loss in their life expectancy. This may also be a factor in Bruxelles-Capitale’s big increase in its mortality level. The pre-pandemic health profile of a district may also be an important factor, in particular its prevalence of chronic conditions or obesity [38, 39]. Moreover, income at the individual level is a factor known to be closely related to health outcomes. Decoster and colleagues [40] showed that there was a significant and negative income gradient in excess mortality during the first wave in Belgium for the elderly. In line with previous finding, Dukhovnov and colleagues [31] showed that Covid-19 mortality rates were 2.58 times higher in the bottom than in the top socio-economically advantaged quintile of counties in USA. In England, communities with high proportion of residents on income support had an increased risk of excess mortality during the first wave of the pandemic [35]. Ginsburgh and colleagues [33] showed that a higher dispersion across incomes within the same French department led to more deaths. Poor housing conditions and higher occupational exposures have been shown to be the most likely mechanisms causing the higher burden of Covid-related mortality for the poor in France [41]. In Italiy, Basellini & Camarda [32] found that regional differences in mortality could be explained by heterogeneity in intergenerational co-residence, number of ICU beds per capita, and timing of the outbreak. These factors may also have played a role in the Belgian context.

Identifying which district characteristics are most associated with excess mortality is beyond the scope of this study, but our estimates can be used by other researchers to perform aggregate analysis on the association between the magnitude of the shock and various risk-factors at the district level. Our study also emphasizes how the uncertainty level that we define and are prepared to tolerate affects the results and their interpretation in the context of small-area analyses. Ideally, various uncertainty thresholds should be presented in the context of small-area research.

Our study is subject to several limitations. First, we did not stratify by gender, for two reasons. On the one hand, we are already working with small death counts, and hence, stratifying further by sex risked increasing noisiness in the data. On the other hand, we were primarily interested in the magnitude of the shock on district-level mortality, which can be measured by the overall life expectancy for both sexes. We expect to see spatial variation in sex-ratios across districts but the range should be limited as the number of Covid-19 deaths are similar for men (49.1%) and women (50.8%) at the national level [27]. However, districts with more care homes may show high sex-ratios since their frail population is characterized by an over-represantation of women. Further research could investigate how the sex ratios of mortality were affected by the pandemic at the small-area level. A second limitation is that we used a linear model for the temporal evolution of the difference across districts with national life expectancy over time. Our explanatory data analysis showed that this linear relationship was appropriate for fitting our data. Instead of using linear model, we could have estimated time series models such as random walks or autoregressive processes. These alternative models would have translated into wider confidence intervals for the loss in life expectancy at the district level. Additionally, forecasts of the difference across districts with national life expectancy in 2020 would have been strongly determined by the last observations. Hence, time series models may have led to implausible counterfactual scenario in small districts where differences with national life expectancy were characterized by high random fluctuation. More suitably, forecasting using a linear model accounts for the long-term trend observed in each district, leading to realistic forecasts.Note however that the linearity assumption is only valid for projection over a very short time horizon of one or two years. If the goal is to obtain a projection over a longer time horizon, we do not advise using our approach and refer instead to the methodology developed by Ševčíková and Raftery (2021) [19]. Our method had the advantage of indirectly accounting for both the temporal trend with respect to national life expectancy and the uncertainty of projections at the district level. Third, period life expectancy as an indicator of the shock on mortality is not perfect. Indeed, it is built on the concept of a hypothetical cohort of individuals experiencing, over their entire life, the age-specific mortality rates seen during 2020. But no real cohort will experience this mortality shock over a long period. As a matter of fact, Belgian life expectancy in 2021 witnessed a bounce-back to its level in 2019 [42]. This suggests that the anticipated harvesting effect [43] in 2020 has been modest, as life expectancy in 2021 did not exceed its level of 2019. In other words, the pandemic did not only advance the deaths of frail individuals precipitately.

## Conclusions

Our study proposed a methodology for assessing the impact of the COVID-19 pandemic in Belgium at the district level. First, using SMR, we looked at the effect of the pandemic on the heterogeneity of mortality across districts. In 2020 there was a widening of its SMR distribution, a sign of higher heterogeneity in mortality levels within Belgium compared to past years. Then, we compared projected and observed life expectancy at the district level. By using the projections as a counterfactual scenario we were able to account for the increasing trend in life expectancy. In estimating the difference between these two measures, we fully accounted for various sources of uncertainty. We showed that the shock has been highly heterogeneous within the country. Some districts experienced a drop of more than two years compared to their projection while others did not experience any significant difference. The uncertainty we are prepared to tolerate has a huge impact on whether we consider a shock to be significant or not. This reinforces the need to carefully account for uncertainty and present various uncertainty levels in the context of small-area analyses.

## Supplementary Information

**Additional file 1** Supplementary materials.

## Data Availability

The data are publicly available from Statbel, the Belgian statistical office, and can be downloaded from https://statbel.fgov.be/en/open-data. The granularity of age groups for the death counts was higher in our data in comparison to what is found on Statbel open data portal. This more disaggregated age specific data was obtained in the context of the CAUSINEQ project, financed by BELSPO.

## Abbreviations

WHO: World Health Organization
IHME: Institute for Health Metrics and Evaluation
NUTS: Nomenclature of Territorial Units for Statistics
BHM: Bayesian hierarchical model
SMR: standardized mortality ratio
