# Supplementary material for: Heterogeneity in subnational mortality in the context of the COVID-19 pandemic: the case of Belgian districts in 2020
Source: Arch Public Health. 2022 May 6;80:130. doi: 10.1186/s13690-022-00874-7 (PMC9073828; doi:10.1186/s13690-022-00874-7)
Supplement: Supplementary file 1 — Additional file 1 Supplementary materials. [file 13690_2022_874_MOESM1_ESM.pdf]

Additional Files

Additional Fig. 1-3 — Linearity of $\alpha_{d,t}$ over time

The figure shows the evolution of $\alpha_{d,t} = e^0_{d,t} - e^0_{Nat,t}$ over time for three districts of various population sizes, namely Liège, Nivelles and Marche-en-Famenne. We added the expected fit from a simple linear regression estimated individually for each district for each year from 1991 to 2019.

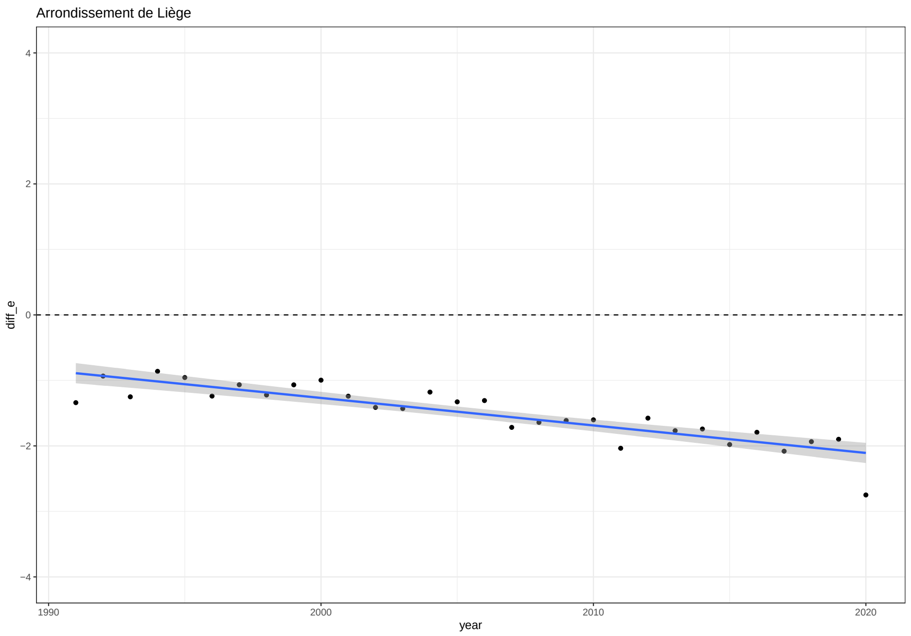

Figure S1 $\alpha_{d,t}$ over time, Liège

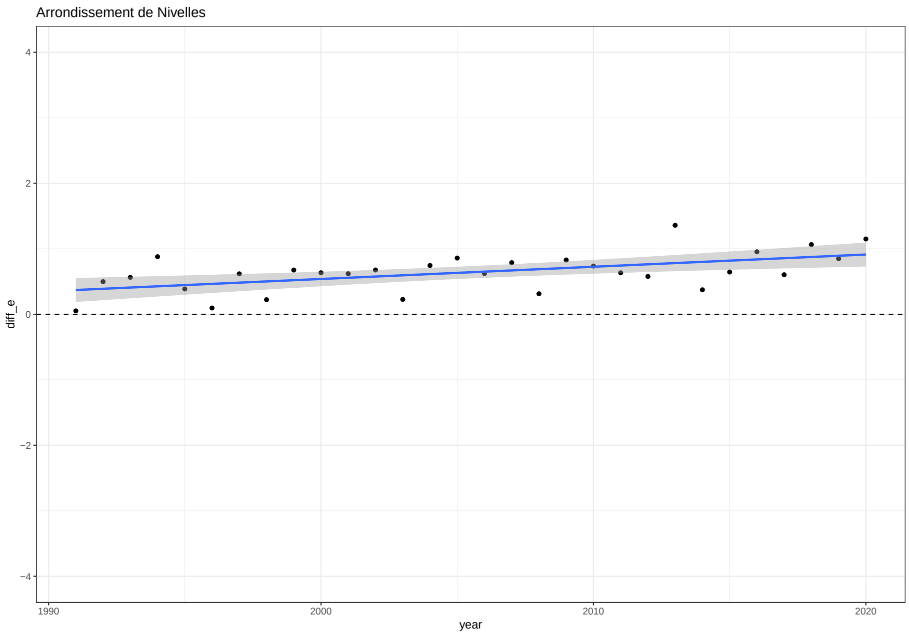

Figure S2 $\alpha_{d,t}$ over time, Nivelles

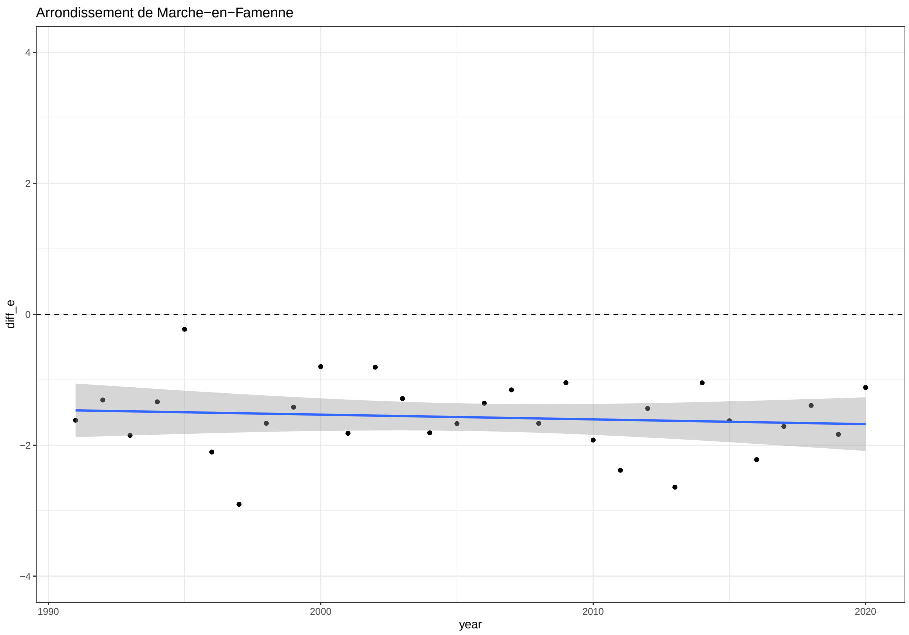

Figure S3 $\alpha_{d,t}$ over time, Marche-en-Famenne

Additional File 1 — Priors in the BHM for $\alpha_{d,t}$

Below are the priors set to the model $\alpha_{d,t} \sim Normal(\beta_{0,d} + \beta_{1,d} \cdot t, \sigma_d)$.

$$\begin{aligned}
\beta_{0,d} &\sim Normal(\mu_{\beta_0}, \sigma_{\beta_0}) \\
\beta_{1,d} &\sim Normal(\mu_{\beta_1}, \sigma_{\beta_1}) \\
\sigma_d &\sim Normal^{+}(\mu_\sigma, \sigma_\sigma)
\end{aligned}$$

where priors for μ_{β_0} and μ_{β_1} are $Normal(0,1)$ and for μ_σ, σ_{β_0}, σ_{β_1}, σ_σ are $Normal^{+}(0,1)$.

Additional Fig. 4 — Prior predictive checks for $\alpha_{d,t}$

The figure below shows the prior predictive distribution of possible $\alpha_{d,t}$ values according to our priors. The range is much higher than what is observed in the data and hence is not informative.

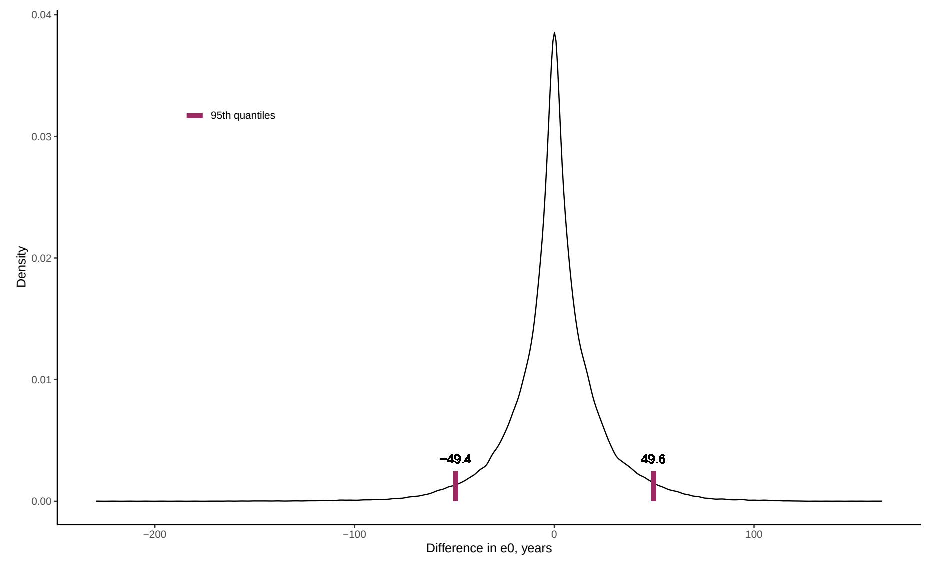

Figure S4 Prior predictive distribution of $\alpha_{d,t}$

Additional Fig. 5 — Posterior predictive checks for $\alpha_{d,t}$

The figure below shows the density overlay between 100 generated posterior draws for $\alpha_{d,t}$ (light blue) and the real $\alpha_{d,t}$ (dark blue). The simulated data does a good job in reproducing the data.

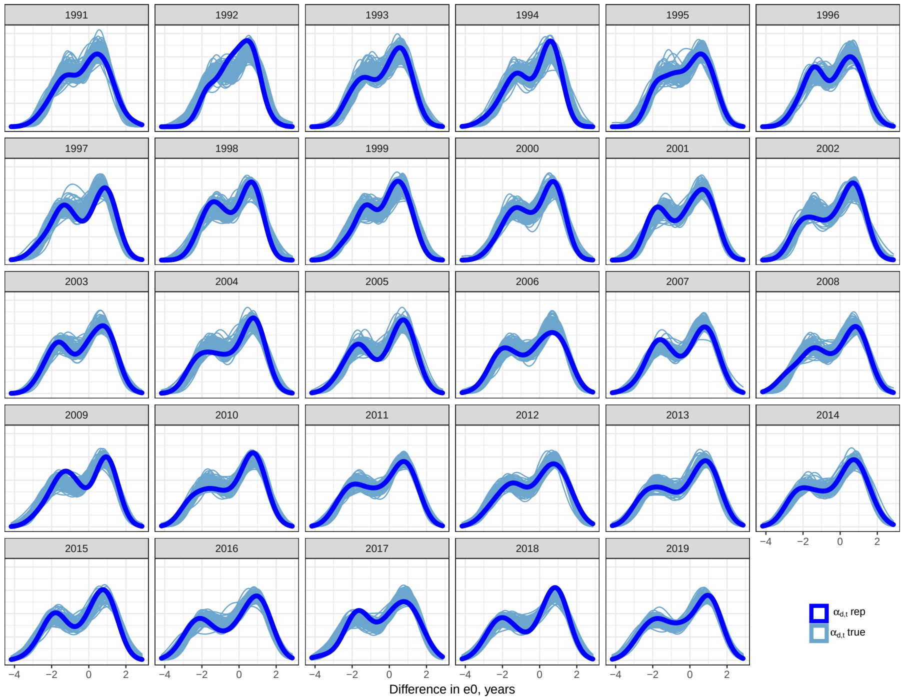

Figure S5 Posterior predictive check, density overlay for 100 generated $\alpha_{d,t}$ draws

Additional Fig.6 — Density of the posterior draws for the standard deviation of standard mortality ratios, 2015–2020

The figure below shows the posterior distribution of the standard deviation of standard mortality ratios over the years considered.

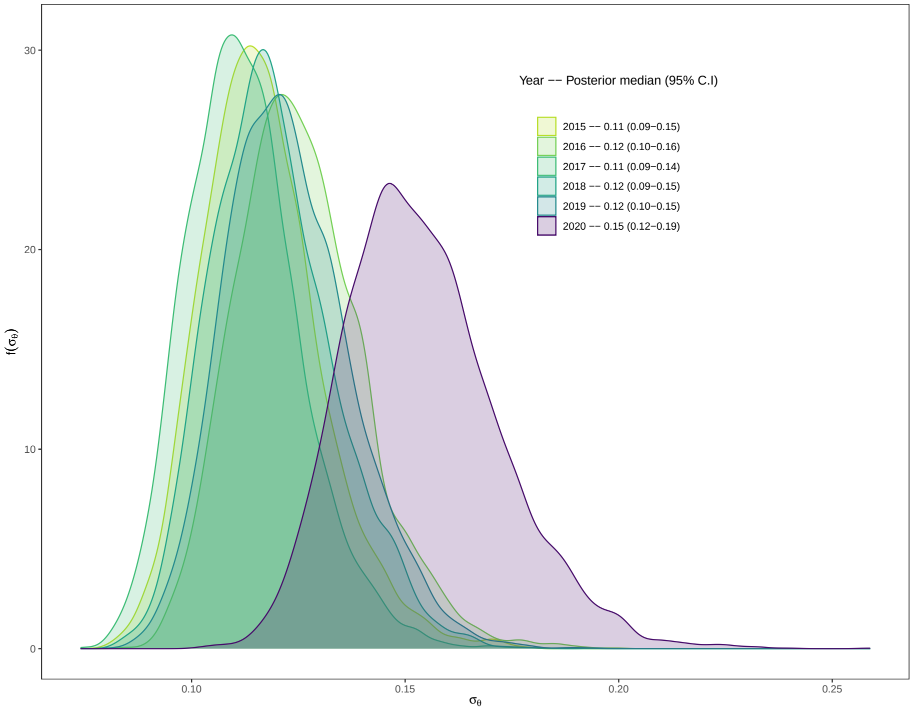

Figure S6 Density of the posterior draws for the standard deviation of SMRs, 2015–2020

Additional Fig. 7 — Density of the simulated standard deviation of Belgian district life-expectancies, 2015-2020

The figure below shows the density of the simulated standard deviation of Belgian district life expectancies over the years considered.

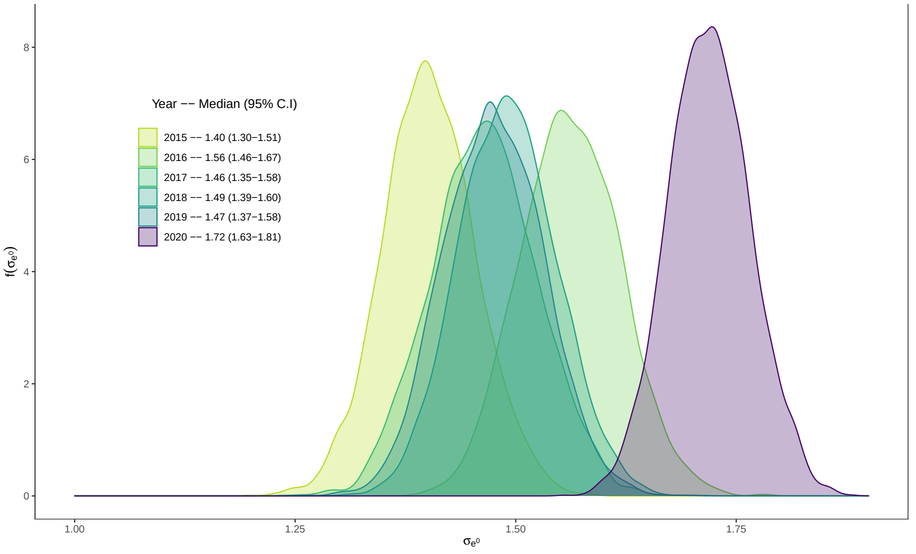

Figure S7 Density of the simulated standard deviation of Belgian district life expectancies, 2015-2020
